# Hereditary angioedema diagnosis evaluation score (HADES): A new clinical scoring system for predicting hereditary angioedema with C1 inhibitor deficiency

**DOI:** 10.1016/j.jacig.2025.100414

**Published:** 2025-01-17

**Authors:** Ricardo Zwiener, Rafael Zamora, Carlos María Galmarini, Laura Brion, Laura Arias, Andrea Pino, Paula Rozenfeld

**Affiliations:** a Allergy and Immunology Department, Hospital Universitario Austral, Pilar, Buenos Aires, Argentina; b Medicus, Buenos Aires, Argentina; c Topazium Artificial Intelligence, Madrid, Spain; d Medical Affairs, Takeda Argentina SA, Buenos Aires, Argentina; e Regional Medical Affairs, Growth and Emerging Markets, Takeda Pharmaceuticals International AG Singapore Branch, Singapore; f Instituto de Estudios Inmunológicos y Fisiopatológicos, Universidad Nacional de La Plata, Consejo Nacional de Investigaciones Científicas y Técnicas (CONICET), Facultad de Ciencias Exactas, Departamento de Ciencias Biológicas, La Plata, Argentina

**Keywords:** Hereditary angioedema, diagnosis, predictive score, clinical variables, C1 inhibitor

## Abstract

**Background:**

Diagnosis of hereditary angioedema (HAE) poses challenges because of its rarity and its overlapping symptoms with allergic and gastrointestinal conditions, resulting in misdiagnosis.

**Objective:**

We developed a predictive score using clinical variables for suspected HAE patients with C1 inhibitor deficiency (HAE-C1INH) to increase suspicion of HAE and thus improve diagnosis.

**Methods:**

The HADES (HAE diagnosis evaluation score) study used a nationwide retrospective cohort of individuals with suspected HAE-C1INH in Argentina. A questionnaire was designed to collect relevant clinical information on possible predictors for HAE. Blood samples were analyzed for C1-INH/C1q levels and C1-INH function. A predictive score was developed from the odds ratios derived from multivariate logistic regression analysis.

**Results:**

The study included 2423 individuals (1642 suspected index cases and 781 family cases). Only patients with confirmed HAE types I or II (n = 499) were included in the final analysis; acquired angioedema/*F12* gene variants were excluded. Eight clinical variables were identified as independent predictors of HAE: age at onset ≤20 years, recurrent limb edema, abdominal pain, vomiting, trauma as a trigger, absence of wheals, family history of angioedema, and recurrent edema lasting ≥24 hours. The predictive score demonstrated favorable performance in identifying HAE cases within the index population (range, 0-18.5), with low scores (1.5-6.5) associated with high sensitivity (100%) and negative predictive value (100%), and high scores (≥15) associated with high specificity (99.4%) and positive predictive value (75.0%).

**Conclusions:**

The predictive HADES offers a simple and efficient method for improving testing for suspicion of HAE by using clinical parameters. Further validation studies are required to confirm its reliability and accuracy.

Hereditary angioedema (HAE) is a rare genetic autosomal-dominant condition characterized by the occurrence of recurrent and unpredictable episodes of edema without wheals affecting the skin, abdomen, and upper airways, which can cause life-threatening complications.^1^^,^^2^ This condition, associated with C1 inhibitor (C1-INH) deficiency (type I), C1-INH dysfunction (type II), or normal-functioning C1-INH (but linked to mutations in the factor XII gene *[HAE-FXII],* plasminogen *[HAE-PLG],* and angiopoetin-1 *[HAE-AGPT1]*, among others), exhibits clinical heterogeneity, with variations in frequency, duration, and severity of episodes. Approximately 40% of individuals with HAE can identify specific triggers that precipitate episodes.^3^ These triggers encompass various factors, including viral and bacterial infections (such as *Helicobacter pylori*), emotional stress (related to work, school, or family), and physical trauma (such as surgical procedures, dental treatments, or accidents), as well as certain medications like angiotensin-converting enzyme inhibitors and contraceptives containing estrogens.^3^, ^4^, ^5^, ^6^, ^7^ However, the diagnosis and management of acute angioedema can be challenging.^8^ In some cases, no underlying cause can be identified despite extensive differential diagnostic evaluation, leading to a diagnosis of idiopathic angioedema.^9^

The rarity of HAE and its overlapping symptoms with other forms of angioedema, coupled with the potential for abdominal attacks to mimic surgical emergencies, often leads to misdiagnosis.^10^, ^11^, ^12^ Zanichelli et al found the most common misdiagnoses to be allergic angioedema (103 of 185) and appendicitis (50 of 185).^10^ Misdiagnoses result in significant delays in receiving the correct diagnosis, thereby depriving patients of timely access to effective, life-saving treatment. The growing awareness of HAE has improved diagnosis rate, but a long delay still exists.^13^ Patients with HAE have a significantly increased risk of premature death due to asphyxiation, in addition to social and economic consequences resulting from untreated HAE.^14^^,^^15^ Surprisingly, studies have shown that patients with a positive family history of HAE are not diagnosed earlier than those without such a history, highlighting the low medical awareness of the disease.^16^

To address these challenges, a group of experts from 10 European countries developed a clinical and laboratory criterion for HAE diagnosis in the Novel Methods for Predicting, Preventing, and Treating Attacks in Patients with Hereditary Angioedema (PREHAEAT) project.^1^ The proposed criteria, developed through expert consensus, comprise 4 major and minor clinical criteria, as well as 3 laboratory criteria that were based on antigenic and functional C1-INH levels and variants in the C1 inhibitor gene that alter protein synthesis and/or function. These criteria represent a critical step toward facilitating the diagnosis of HAE in clinical practice and provide a valuable tool for health care professionals in their daily patient care.

To date, few clinical predictive scores for suspected HAE cases have been described,^17^^,^^18^ but nevertheless, predictive scores for HAE are not commonly utilized in routine clinical settings. Therefore, the objective of this study was to develop an accessible, specific, and sensitive predictive tool for HAE with C1-INH deficiency (HAE-C1INH) diagnosis in patients with angioedema and their relatives that is based on clinical data before laboratory confirmation.

## Methods

### Study design and population

**Fig 1:**
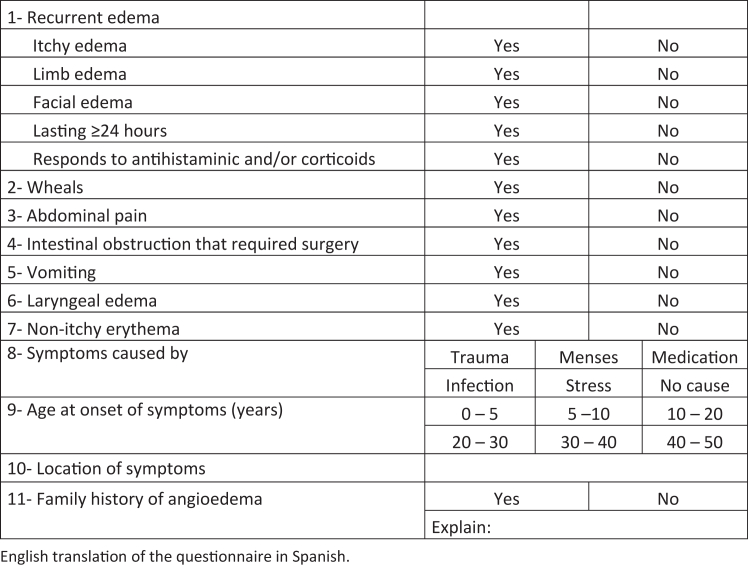
Guidance questionnaire for suspected HAE cases. English translation of questionnaire in Spanish.

The HADES (HAE diagnosis evaluation score) study used a cross-sectional nationwide approach, focusing on individuals with suspected HAE and their relatives. Allergists and immunologists specializing in HAE from 16 centers across Argentina recruited cases of clinically suspected HAE as suspected index cases or family cases. Suspected index cases occurred in individuals whose HAE was initially detected at the consultation with the allergist/immunologist as presenting with symptoms compatible with HAE. Family cases are relatives of HAE-confirmed index cases who were invited to medical consultations for family tree analysis. Family tree analysis was supported by a Takeda patient-support program through genetic counseling of the families and testing at-risk relatives as determined by autosomal inheritance of HAE. A questionnaire was developed by allergy specialists and experts from the University of La Plata, Argentina (UNLP), that was based on their experience in treating patients with HAE and a thorough literature review (Fig 1, and see Fig E1 in the Online Repository available at www.jaci-global.org). The questionnaire was designed to collect as much relevant information as possible on symptom characteristics, age at onset, family history, and triggers of symptoms in a user-friendly format. The questionnaire was completed by physicians for both suspected index cases and family cases. The questionnaire, a prerequisite for sample analysis, had to be submitted by the attending physician along with the patient’s sample for confirmatory genetic testing. All samples were processed and analyzed at Diel’s laboratory at the Instituto de Estudios Inmunológicos y Fisiopatológicos (IIFP), UNLP/CONICET. The analysis included all samples submitted for analysis to Diel’s laboratory from 2014 to 2022. Questionnaires that had missing data (4.8%) were not included in the analysis.

The study protocol was approved by the ethics committees or institutional review board of the UNLP, and all patients provided informed consent to use their clinical data for scientific purposes.

### Sample processing and HAE diagnosis

All samples underwent both quantitative and functional C1-INH tests. The diagnosis of HAE was established when functional C1-INH levels fell below 70%. Quantitative analysis of C1-INH levels was conducted to differentiate between type I and type II HAE. Normal C1q levels were used to rule out acquired angioedema.

Venous blood was extracted (10 mL) and dispensed into 2 tubes with citrate (2 mL each), 1 dry tube (4 mL), to obtain plasma and serum, respectively, and 1 tube with EDTA (1 mL). Samples were shipped on dry ice and stored at −80°C until use. Radial immunodiffusion was used to quantify C1-INH and C1q in serum only in index cases (Binding Site Group), and functional C1-INH in plasma was determined by a colorimetric method (Siemens Healthcare Diagnostics Products). When requested, genetic analysis to identify pathogenic variants c.983C>A (p.Thr328Lys) and c.983C>G (p.Thr328Arg) in the *F12* gene was performed by Sanger sequencing of exon 9 of the *F12* gene.

### Predictive score development and statistical analysis

A multivariate logistic regression analysis was conducted to identify variables significantly associated with HAE diagnosis among symptomatic patients and family members. Clinical variables derived from the questionnaire with statistical significance (*P* < .2) in the univariate analysis were incorporated into the logistic regression model, using a backward stepwise approach to derive the final predictors. Independent predictive factors that retained statistical significance in the final logistic regression analysis were used to compute the predictive score. Model performance was evaluated for goodness of fit and calibration using the Hosmer-Lemeshow test statistic. The prognostic model was internally validated by bootstrapping procedure with over 1000 samples. The primary variables, including sensitivity, specificity, positive prediction value (PPV), negative prediction value (NPV), and percentage correctly classified for the presence of HAE, were estimated on the basis of the prediction score using a probability threshold/cutoff of 0.50. This cutoff value was selected on the basis of the high individual variability and potential severity of HAE. The area under the receiver operating characteristic curve was used to measure the performance of logistic regression models. Youden indices were calculated to determine the sensitivity and specificity of the score cutoffs (Youden index = sensitivity + specificity − 1).

Only patients with confirmed HAE type I or II (n = 499) were included in the analysis. Patients with acquired angioedema (n = 12) or variants in the *F12* gene (n = 47) were excluded. HAE-FXII patients were excluded because they were overrepresented in this cohort (8.5% cases); these cases represent <2% in the real world. We did not include the location of the patients’ edema (Fig 1, item 10) because of the open-ended nature of the response. Additionally, the age at onset of symptoms was not analyzed in family cases because of the inherent nature of these cases, which were studied because a family member was diagnosed instead of relying on individual symptom recall.

Continuous variables were reported as medians (with interquartile ranges [IQRs]) and categorical variables as frequencies and proportions. *P* values are presented for 2-sided testing of hypotheses (level of significance at 5%), unless otherwise specified. All data were analyzed by SPSS v29 software (IBM, Armonk, NY).

## Results

The study enrolled 1642 patients suspected of having HAE (suspected index cases) and 781 relatives of suspected index cases who were later confirmed as having HAE (family cases). Among the 1642 suspected index cases, 158 (9.6%) received a confirmed diagnosis of HAE (132 type I, 26 type II). Similarly, among the family cases, 341 individuals (43.6%) were diagnosed with HAE (313 type I, 28 type II). We used family tree analysis to conclude that the resulting 499 HAE cases belonged to 163 unique families. Additionally, we identified 12 patients with acquired angioedema and 47 patients with variants in the *F12* gene (only 9 of whom were in the suspected index case cohort); however, these cases were excluded from the analysis. Baseline characteristics of the overall population (suspected index cases and family cases) as well as for HAE versus non-HAE groups are presented in Table I. Of the patients diagnosed with HAE, confirmed index cases had a median (IQR) age of 41 (25-55) years with a predominantly female majority (64.4%). Confirmed family patients had a median (IQR) age of 27 (13-42) years, with female participants constituting the majority (54.0%). Among all suspected index cases, 906 (55.2%) presented with an age at onset ≤20 years, and 199 (12.1%) had a family history of angioedema (but without a confirmed diagnosis of HAE within the family). Clinical symptoms were frequently reported among the suspected index population, with facial edema being the most frequently reported symptom (77.9%), followed by limb edema (52.5%) and abdominal pain (45.1%). However, the presence of recurrent facial and limb edema as well as abdominal pain was similar in HAE-confirmed index cases (77.2%, 72.8%, and 73.4%, respectively). Among the known triggers, trauma was the most commonly reported driver for symptoms in both suspected index cases and family cases with confirmed HAE diagnosis. Nevertheless, 884 suspected index cases (53.1%) could not identify any triggers for symptoms (Table I).

**Table I:** Baseline characteristics of study population

Characteristic	Suspected index cases				Family cases			
	Total population (N = 1642)	No HAE (n = 1484)	HAE (n = 158)	*P* value	Total population (N = 781)	No HAE (n = 440)	HAE (n = 341)	*P* value
Age (years), median (IQR)	33 (16-47)	33 (15-47)	41 (25-55)	<.001	25 (12-42)	24 (11-42)	27 (13-42)	.192
Female gender	1021 (62.2)	919 (61.9)	102 (64.4)	.517	435 (55.7)	251 (57.0)	184 (54.0)	.389
Age at onset ≤20 years	906 (55.2)	795 (53.6)	111 (70.3%)	<.001	NA	NA	NA	
Family history of angioedema	199 (12.1)	154 (10.4)	45 (28.5)	<.001	NA	NA	NA	
Recurrent edema lasting ≥24 hours	530 (32.3)	449 (30.3)	81 (51.3)	<.001	139 (17.8)	14 (3.2)	125 (36.7)	<.001
Recurrent itchy edema	605 (36.8)	569 (38.3)	36 (22.8)	<.001	70 (9)	12 (2.7)	58 (17)	<.001
Recurrent limb edema	862 (52.5)	747 (50.3)	115 (72.8)	<.001	212 (27.1)	34 (7.7)	178 (52.2)	<.001
Recurrent facial edema	1279 (77.9)	1157 (78)	122 (77.2)	.829	199 (25.5)	35 (8)	164 (48.1)	<.001
Recurrent edema that responds to antihistamines/corticosteroids	209 (12.7)	199 (13.4)	10 (6.3)	.011	15 (1.9)	0	15 (4.4)	<.001
Laryngeal edema	554 (33.7)	484 (32.6)	70 (44.3)	.003	88 (11.3)	11 (2.5)	77 (22.6)	<.001
Wheals	730 (44.5)	686 (46.2)	44 (27.8)	<.001	90 (11.5)	37 (8.4)	53 (15.5)	.002
Abdominal pain	740 (45.1)	624 (42)	116 (73.4)	<.001	231 (29.6)	60 (13.6)	171 (50.1)	<.001
Intestinal obstruction that required surgery	53 (3.2)	35 (2.4)	18 (11.4)	<.001	27 (3.5)	5 (1.1)	22 (6.5)	<.001
Vomiting	232 (14.1)	168 (11.3)	64 (40.5)	<.001	119 (15.2)	27 (6.1)	92 (27)	<.001
Nonitchy erythema	465 (28.3)	408 (27.5)	57 (36.1)	.023	85 (10.9)	18 (4.1)	67 (19.6)	<.001
Previous receipt of NSAIDs or ACE inhibitors	101 (6.2)	94 (6.3)	7 (4.4)	.344	4 (0.5)	1 (0.2)	3 (0.9)	.223
Symptoms triggered by								
No cause	884 (53.1)	823 (55.5)	59 (37.3)		150 (49.2)	90 (75)	60 (32.4)	
Trauma	209 (12.7)	150 (10.1)	59 (37.3)		92 (30.2)	10 (8.3)	82 (44.3)	
Menses	38 (2.3)	37 (2.5)	1 (0.6)		5 (1.6)	1 (0.8)	4 (2.2)	
Medication	64 (3.9)	63 (4.2)	1 (0.6)		4 (1.3)	1 (0.8)	3 (1.6)	
Infection	30 (1.8)	29 (2.0)	1 (0.6)		2 (0.7)	0	2 (0.7)	
Stress	419 (25.5)	382 (25.7)	37 (23.4)		52 (17)	18 (15)	34 (18.4)	

Data are presented as nos. (%) unless otherwise indicated.

*ACE,* Angiotensin-converting enzyme; *NA*, not applicable; *NSAID,* nonsteroidal anti-inflammatory drug.

In the univariate analysis of the suspected index cases, HAE was significantly associated with older age, age at onset ≤20 years, recurrent edema lasting ≥24 hours, recurrent limb edema, laryngeal edema, abdominal pain, intestinal obstruction that required surgery, vomiting, nonitchy erythema and less itchy edema, family history of angioedema, lower incidence of recurrent edema responsive to antihistamines/corticosteroids, and lower incidence of wheals. In the family cases, HAE was significantly associated with recurrent edema lasting ≥24 hours, recurrent limb edema, recurrent facial edema, laryngeal edema, itchy edema, recurrent edema responding to antihistamines/corticosteroids, wheals, abdominal pain, intestinal obstruction requiring surgery, vomiting, and nonitchy erythema (Table I).

**Fig 2:**
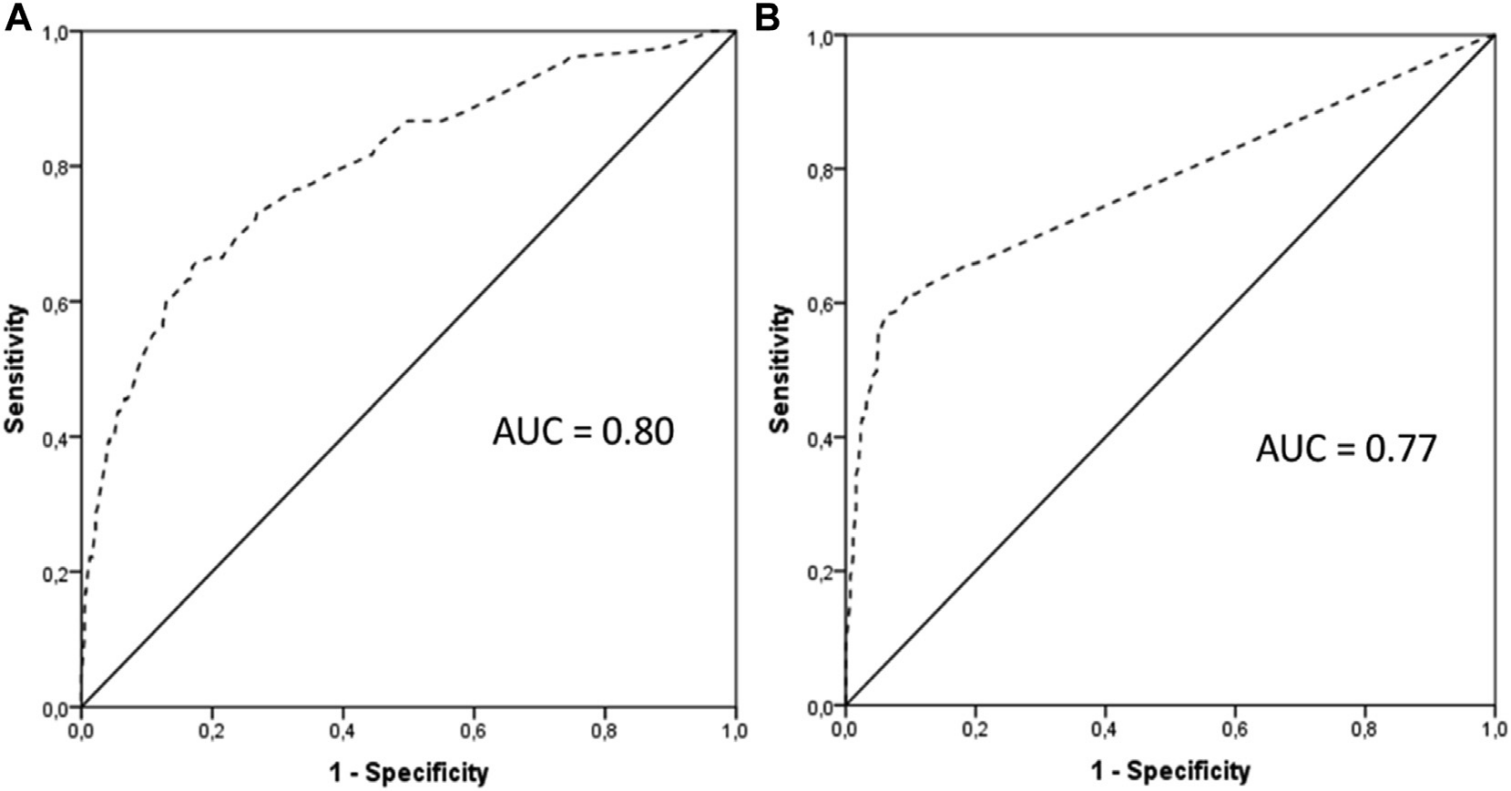
Receiver-operating characteristic curves. **(A)** Suspected index cases. **(B)** Family cases. *AUC,* Area under curve.

Logistic regression analysis identified that age at onset ≤20 years, abdominal pain, recurrent limb edema, absence of wheals, family history of angioedema, vomiting, and trauma as a trigger for symptoms were independent predictors of HAE in suspected index cases. In family cases, the independent predictors of HAE identified were abdominal pain, trauma as a symptom trigger, recurrent limb edema, and recurrent edema lasting ≥24 hours (Table II). The area under the receiver operating characteristic curve (95% confidence interval) for the suspected index cases model was 0.80 (0.76-0.84) and 0.77 (0.74-0.81) for the family cases model (Fig 2). The internal validation of these variables was checked by bootstrapping with 1000 random samples, which demonstrated consistent beta coefficients across the resampled datasets (see Tables E1 and E2 in the Online Repository available at www.jaci-global.org). The HADES was calculated according to the odds ratio obtained from the logistic regression analysis. To enhance clarity and facilitate practical application, each odds ratio was rounded to the nearest integer or 0.5, as appropriate. The resulting HADES ranged from 0 to 18.5 in suspected index cases and 0 to 15 in family cases (Table III).

**Table II:** Logistic regression analysis of predictors of HAE

Characteristic	β	Odds ratio (95% confidence interval)	*P* value
Suspected index cases∗			
Age at onset ≤20 years	0.537	1.71 (1.15-2.53)	.007
Recurrent limb edema	0.89	2.44 (1.63-3.66)	<.001
Abdominal pain	0.635	1.88 (1.24-2.87)	.003
Family history of angioedema	1.128	3.09 (1.97-4.83)	<.001
Vomiting	1.211	3.35 (2.19-5.12)	<.001
Trauma as trigger of symptoms	1.262	3.53 (2.34-5.32)	<.001
Absence of wheals	0.927	2.52 (1.69-3.76)	<.001
Family cases†			
Recurrent limb edema	1.506	4.51 (2.27-7.38)	<.001
Recurrent edema ≥24 hours	1.806	6.08 (3.24-11.42)	<.001
Abdominal pain	0.633	1.88 (1.21-2.93)	.005
Trauma as trigger of symptoms	0.921	2.51 (1.14-5.53)	.022

Hosmer-Lemeshow ∗*P* = .43 and †*P* = .13.

**Table III:** Summary of point scores in diagnostic rule

Characteristic	Point score
Suspected index cases	
Age at onset ≤20 years	1.5
Abdominal pain	2
Recurrent limb edema	2.5
Absence of wheals	2.5
Family history of angioedema	3
Vomiting	3.5
Trauma as trigger of symptoms	3.5
Range of score	0-18.5
Family cases	
Abdominal pain	2
Trauma as trigger of symptoms	2.5
Recurrent limb edema	4.5
Recurrent edema lasting ≥24 hours	6
Range of score	0-15

To address the challenge of the limited number of events (HAE cases) in certain score categories (see Figs E2 and E3 in the Online Repository available at www.jaci-global.org), we opted to group the HADES, ensuring sufficient events within each category for robust analysis (see Tables E3 and E4 in the Online Repository). The categories for grouping were manually determined considering the positivity rates for HAE within each category. We approached this grouping with the logic that higher scores were associated with higher positivity rates. Consequently, the resulting categories (points for suspected index cases: A, 0; B, 1.5-6.5; C, 7-9; D, 9.5-11; E, 11.5-14.5; and F, 15-18.5; and points for family cases: A, 0; B, 2-4.5; C, 6-7; and D, ≥8), although asymmetric because of the different distribution of events and performance, were created to maximize the discriminative power of the HADES, ensuring a meaningful differentiation between various levels of diagnostic probability. Table IV summarizes the performance of the HADES in identifying patients with HAE across multiple score levels. None of the 56 suspected index subjects with 0 points was diagnosed with HAE, indicating that a minimum of 1.5 points has 100% sensitivity and NPV. Higher scores decrease sensitivity but increase specificity and accuracy (99.39% and 91.47%, respectively, for scores of ≥15 points). For family cases, the sensitivity is lower; the presence of at least 2 points has a sensitivity of 65.69% and a NPV of 75.26%; nevertheless, specificity and PPV are higher in higher scores (97.5% and 92.72% respectively, for score of ≥8 points).

**Table IV:** Performance of HADES category scores in screening for HAE cases

Category	Score	Total no. of subjects	No. of HAE cases	Sensitivity (%)	Specificity (%)	PPV (%)	NPV (%)	Correctly classified (%)
Suspected index cases								
A	0	56	0	NA	NA	NA	NA	NA
B	1.5-6.5	1062	42	100	3.77	9.96	100	13.03
C	7-9	275	29	73.42	72.51	22.14	96.24	72.59
D	9.5-11	126	25	55.06	89.08	34.94	94.9	85.81
E	11.5-14.5	87	35	39.24	95.89	50.41	93.68	90.44
F	15-18.5	36	27	17.09	99.39	75	91.84	91.47
Family cases								
A	0	473	117	NA	NA	NA	NA	NA
B	2-4.5	91	34	65.69	80.91	72.73	75.26	74.26
C	6-7	66	50	55.72	93.86	87.56	73.23	77.21
D	≥ 8	151	140	41.06	97.50	92.72	68.10	72.86

Categories refer to points for suspected index cases: A, 0; B, 1.5-6.5; C, 7-9; D, 9.5-11; E, 11.5-14.5; and F, 15-18.5; and points for family cases: A, 0; B, 2-4.5; C, 6-7; and D, ≥8.

*NA,* Not applicable.

## Discussion

In this study, we developed a risk prediction model for the diagnosis of HAE-C1INH in symptomatic patients suspected of having this disease, as well as in family members of HAE-C1INH patients, using a simple questionnaire of symptoms and clinical characteristics. Two scoring systems were created to classify these 2 models—suspected index cases and family cases—into 7 and 4 risk categories, respectively.

Our logistic regression analysis identified 7 statistically significant predictors of HAE in suspected index cases, including age at onset ≤20 years, recurrent limb edema, abdominal pain, vomiting, trauma as a trigger of symptoms, absence of wheals, and family history of angioedema. In family cases, the statistically significant predictors were recurrent limb edema, recurrent edema lasting ≥24 hours, abdominal pain, and trauma as a trigger of symptoms. The HADES demonstrated good performance in identifying HAE in both suspected index cases and family cases, with low scores associated with high sensitivity and NPV, and high scores associated with high specificity and PPV. None of the suspected index cases with a score of 0 was HAE. Higher HADES generally indicate an increased likelihood of HAE, and the cutoff point can be customized for specific applications. For example, a questionnaire cutoff value of ≥1.5 points yields a sensitivity and NPV of 100%, but very low specificity and PPV for suspected index cases. Conversely, a cutoff of ≥11.5 points yields a specificity of 95.89% and a sensitivity of 39.24% (99.39% and 17.09%, respectively, for a cutoff of ≥15). The performance of HADES for family cases is lower. This may be explained by the fact that family cases were tested because they were relatives of patients with HAE rather than because of symptoms. Another possible explanation is that as a result of our better knowledge of the disease, family members of those affected may visit the doctor earlier and therefore have fewer symptoms. To maximize sensitivity and capture as many potential HAE cases as possible, a score corresponding to high sensitivity with low specificity can be considered.^19^

HAE is commonly misdiagnosed, probably because of poor medical awareness of the disease.^10^, ^11^, ^12^, ^13^^,^^20^^,^^21^ The rarity of the condition, the heterogeneity of its affected population, and the overlap in symptoms with other, more common diseases make diagnosing HAE challenging.^16^^,^^21^, ^22^, ^23^ A 2013 analysis of Icatibant Outcome Survey data found a median diagnostic delay in patients with HAE across 8 European countries of 8.5 years.^16^ Previous nationwide surveys in Spain and Denmark reported mean delays in diagnosis of 13.1 and 16.3 years, respectively.^21^^,^^22^ Furthermore, in an international web-based survey, 313 patients with HAE reported visiting an average of 4.4 physicians over an average of 8.3 years before receiving an accurate HAE diagnosis.^24^ Possible explanations for the differences in diagnostic delay between countries include awareness of HAE among physicians, patient selection bias, and demographic differences among patient populations. The clinical (potentially fatal), social, and economic consequences of this lag in HAE diagnosis have been well described.^11^^,^^12^ Remarkably, the average age at diagnosis in suspected index cases of HAE in our population was 41 years, which is 17 years later than the average age at diagnosis in the European population.^16^ However, 70% of HAE patients in our cohort reported an age at onset of ≤20 years. This lengthy delay highlights the significant risk faced by these patients as a result of the lack of medical awareness of this condition, but it also represents an opportunity to use the score not only to improve diagnosis but also to increase awareness and knowledge of the disease within the medical community and among patients.

It is well established that patients with a confirmed family history of HAE are significantly less likely to receive misdiagnoses, presumably because of consideration of their family history in the differential diagnosis. Furthermore, in families with known HAE-C1INH, it is important to mention that first-degree relatives, whether symptomatic or asymptomatic, should be screened for C1-INH (preferably functional) and C4 levels at their earliest convenience.^25^ Nevertheless, it is noteworthy that nearly half of patients with a family history of HAE still receive misdiagnoses,^10^ and previous studies have shown that they are not diagnosed earlier than those without a family history.^16^ In this study, the median age at diagnosis of HAE in family cases was 27 years, which was 14 years earlier than the median age at diagnosis in the suspected index population. Furthermore, 341 (68%) of 499 patients with HAE in this study were family cases, consistent with the 75% reported in previous studies.^26^^,^^27^ However, 45 additional subjects in the suspected index case group reported a family history of angioedema but were tested primarily because of their symptoms, not family history. These findings suggest an opportunity to improve diagnosis by encouraging family members to seek diagnosis and by providing support to primary care physicians to facilitate the diagnostic process.

Few previous scores for HAE diagnosis have been developed.^1^^,^^17^^,^^18^ Aimed as an aid tool in the emergency department, the rapid triage tool developed by Betschel et al includes only 3 predictive variables (absence of urticaria, recurrent abdominal pain/swelling, and lack of response to allergic-directed therapy) and was validated in a small retrospective sample of 107 patients (66 with HAE and 41 without).^17^ A more robust yet complex tool was developed by Shams et al using 25 variables (including laboratory and genetic testing results) to be used on electronic health records to provide an early warning flag to identify patients at risk for HAE.^18^ In turn, the HADES represents a comprehensive tool to establish a clinical-only scoring system aimed at enhancing suspicion of HAE and facilitating a prompt diagnosis. The strength of HADES is the relatively large real-world nationwide database used (2423 subjects in total), along with the inclusion of symptomatic negative cases and nearly 500 HAE-confirmed patients. Furthermore, the confirmed HAE cases we used for the predictive score demonstrated comparable clinical characteristics as described elsewhere, thus minimizing the likelihood of potential country-specific biases.^2^^,^^28^ Because most patients have multiple risk factors in various combinations, a multifactorial risk prediction scheme is likely to be more effective in predicting HAE. Additionally, this model reliably predicts risk using only common, clinically available parameters and uses a simple scoring system that ensures ease of widespread use. Therefore, risk assessment that is based on this model can lead to prompt interventions that have the potential to increase HAE diagnosis rates and reduce misdiagnosis, thereby facilitating close follow-up and aggressive clinical management if necessary. Importantly, providing ongoing education to physicians who are most likely to encounter patients with HAE can significantly reduce the rate of misdiagnosis and minimize delays in receiving an accurate diagnosis and appropriate treatment. Although several published studies and guidelines have increased the awareness of HAE in the scientific community,^15^^,^^21^^,^^29^, ^30^, ^31^, ^32^ it remains underdiagnosed, and misdiagnoses are still frequent.^11^^,^^12^ To address this situation, it is essential to implement active screening of family members, to ensure access to effective and well-tolerated treatment options, and to educate patients and their families about the benefits of diagnosis and treatment. These measures are crucial to improve this unsatisfactory situation.

This observational study has several limitations. The first caveat is the absence of external validation of HADES in populations and settings different from the derivation cohort. Alternatively, a methodological approach, such as a resampling method based on a random number generator for sample division, to build the model on one subset and validate it on another would reduce the risk of relying on sample-specific characteristics. However, given the limited number of events, this approach was not feasible, which may have led to the creation of a less robust model prone to overfitting and unstable estimates. The second limitation was the inherent characteristics of the cohort (low prevalence of HAE and thus low number of events). This made it necessary to group scores because of the lack of sufficient events in some categories. Additionally, because the questionnaire was originally designed for a different purpose, there may have been features that were not collected and could have affected the diagnosis of the disease. Another potential limitation is the possibility of patient recall bias: the onset of symptoms occurred several years before the questionnaire was completed. This bias may explain the low rate of response to antihistamines and/or corticosteroids reported in the group without HAE in the suspected index cases or the higher rate of itchy edema in confirmed-HAE family cases. Also, laboratory results were not included in the development of the HADES because the aim of this predictive score is to rely solely on clinical manifestations and family history. Incorporating C4 laboratory results could potentially improve the accuracy of the score. The development of the HADES focused only on patients with HAE-C1INH, so future efforts should take into consideration all forms of HAE. At any rate, we consider the development of HADES to be a valuable step forward in improving the visibility and awareness of this disease. We believe that subsequent validation of the score and incorporation of more powerful tools for analysis, such as artificial intelligence, are imperative for diagnosing the condition at earlier ages.

In summary, we developed, a simple, specific, and sensitive predictive score for HAE diagnosis in patients with angioedema that is based on clinical data before laboratory confirmation using a nationwide cohort that included nearly 500 HAE-C1INH patients. The use of a clinical score can potentially improve HAE diagnosis and increase awareness among patients and medical professionals, ultimately resulting in earlier detection and improved disease management. Further validation studies using external and/or prospective data sources are necessary to confirm the reliability and accuracy of HADES.

**Key messages**

• The diagnosis of HAE is complex because of its rarity and its overlapping symptoms with allergic and gastrointestinal conditions. This often results in misdiagnosis and treatment delays, thus emphasizing the need for increased clinical awareness.
• We developed a predictive scoring system, HADES, that uses clinical variables. This innovation aims to increase suspicion of HAE-C1INH, thereby enhancing diagnostic accuracy and ultimately advancing patient care.
• The predictive HADES is a straightforward and effective method for increasing the suspicion of HAE using clinical parameters.

## Disclosure statement

Supported by Takeda Argentina SA.

Disclosure of potential conflict of interest: R. Zwiener has been a speaker for Shire/Takeda, CSL Behring, Novartis, Sanofi and Panalab, and Pint Pharma; advisor for Shire/Takeda, 10.13039/100008322CSL Behring, Abbvie, Bago, Kalvista, Pint Pharma, and Pharvaris; and has received financial support for research for Shire/Takeda and Sanofi. R. Zamora has been advisor for 10.13039/100004325AstraZeneca and reports grant funding from Novartis. C. M. Galmarini is an employee and holds stocks in Topazium Artificial Intelligence. L. Brion is an employee at Takeda Argentina SA. L. Arias was an employee at Takeda Argentina SA during the development of this project and is currently employed by Productos Roche SAQ e I Pharma, Buenos Aires, Argentina. A. Pino is an employee of Takeda Pharmaceuticals International AG Singapore Branch and holds stock in Takeda. P. Rozenfeld has received consulting fees and grants from Takeda and Amicus.

## References

[1] Agostoni A., Aygören-Pürsün E., Binkley K.E., Blanch A., Bork K., Bouillet L. (2004). Hereditary and acquired angioedema: problems and progress: proceedings of the Third C1 Esterase Inhibitor Deficiency Workshop and beyond. J Allergy Clin Immunol.

[2] Bork K., Meng G., Staubach P., Hardt J. (2006). Hereditary angioedema: new findings concerning symptoms, affected organs, and course. Am J Med.

[3] Caballero T., Maurer M., Longhurst H.J., Aberer W., Bouillet L., Fabien V. (2016). Triggers and prodromal symptoms of angioedema attacks in patients with hereditary angioedema. J Investig Allergol Clin Immunol.

[4] Caballero T., Farkas H., Bouillet L., Bowen T., Gompel A., Fagerberg C. (2012). International consensus and practical guidelines on the gynecologic and obstetric management of female patients with hereditary angioedema caused by C1 inhibitor deficiency. J Allergy Clin Immunol.

[5] Bouillet L., Longhurst H., Boccon-Gibod I., Bork K., Bucher C., Bygum A. (2008). Disease expression in women with hereditary angioedema. Am J Obstet Gynecol.

[6] Saule C., Boccon-Gibod I., Fain O., Kanny G., Plu-Bureau G., Martin L. (2013). Benefits of progestin contraception in non-allergic angioedema. Clin Exp Allergy.

[7] Zotter Z., Csuka D., Szabó E., Czaller I., Nébenführer Z., Temesszentandrási G. (2014). The influence of trigger factors on hereditary angioedema due to C1 inhibitor deficiency. Orphanet J Rare Dis.

[8] Hahn J., Hoffmann T.K., Bock B., Nordmann-Kleiner M., Trainotti S., Greve J. (2017). Angioedema—an interdisciplinary emergency. Dtsch Arztebl Int.

[9] Lumry W.R. (2013). Overview of epidemiology, pathophysiology, and disease progression in hereditary angioedema. Am J Manag Care.

[10] Zanichelli A., Longhurst H.J., Maurer M., Bouillet L., Aberer W., Fabien V. (2016). Misdiagnosis trends in patients with hereditary angioedema from the real-world clinical setting. Ann Allergy Asthma Immunol.

[11] Wong J.C.Y., Cheong N., Lau C.S., Li P.H. (2022). Prevalence and impact of misdiagnosed drug allergy labels among patients with hereditary angioedema. Front Allergy.

[12] Cao Y., Liu S., Zhi Y. (2021). Recurrent and acute abdominal pain as the main clinical manifestation in patients with hereditary angioedema. Allergy Asthma Proc.

[13] Zanichelli A., Magerl M., Longhurst H.J., Aberer W., Caballero T., Bouillet L. (2018). Improvement in diagnostic delays over time in patients with hereditary angioedema: findings from the Icatibant Outcome Survey. Clin Transl Allergy.

[14] Bork K., Hardt J., Witzke G. (2012). Fatal laryngeal attacks and mortality in hereditary angioedema due to C1-INH deficiency. J Allergy Clin Immunol.

[15] Lumry W.R., Castaldo A.J., Vernon M.K., Blaustein M.B., Wilson D.A., Horn P.T. (2010). The humanistic burden of hereditary angioedema: impact on health-related quality of life, productivity, and depression. Allergy Asthma Proc.

[16] Zanichelli A., Magerl M., Longhurst H., Fabien V., Maurer M. (2013). Hereditary angioedema with C1 inhibitor deficiency: delay in diagnosis in Europe. Allergy Asthma Clin Immunol.

[17] Betschel S., Avilla E., Kanani A., Kastner M., Keith P., Binkley K. (2020). Development of the hereditary angioedema rapid triage tool. J Allergy Clin Immunol Pract.

[18] Shams M., Laney D.A., Jacob D.A., Yang J., Dronen J., Logue A. (2022). Validation of a suspicion index to identify patients at risk for hereditary angioedema. J Allergy Clin Immunol Glob.

[19] Power M., Fell G., Wright M. (2013). Principles for high-quality, high-value testing. Evid Based Med.

[20] Caballero T., Baeza M.L., Cabañas R., Campos A., Cimbollek S., Gómez-Traseira C. (2011). Consensus statement on the diagnosis, management, and treatment of angioedema mediated by bradykinin. Part II. Treatment, follow-up, and special situations. J Investig Allergol Clin Immunol.

[21] Maurer M., Magerl M., Betschel S., Aberer W., Ansotegui I.J., Aygören-Pürsün E. (2022). The international WAO/EAACI guideline for the management of hereditary angioedema—the 2021 revision and update. Allergy.

[22] Roche O., Blanch A., Caballero T., Sastre N., Callejo D., López-Trascasa M. (2005). Hereditary angioedema due to C1 inhibitor deficiency: patient registry and approach to the prevalence in Spain. Ann Allergy Asthma Immunol.

[23] Bygum A. (2009). Hereditary angio-oedema in Denmark: a nationwide survey. Br J Dermatol.

[24] Lunn M.L., Santos C.B., Craig T.J. (2010). Is there a need for clinical guidelines in the United States for the diagnosis of hereditary angioedema and the screening of family members of affected patients?. Ann Allergy Asthma Immunol.

[25] Farkas H., Martinez-Saguer I., Bork K., Bowen T., Craig T., Frank M. (2017). International consensus on the diagnosis and management of pediatric patients with hereditary angioedema with C1 inhibitor deficiency. Allergy.

[26] Busse P.J., Christiansen S.C. (2020). Hereditary angioedema. N Engl J Med.

[27] Pappalardo E., Cicardi M., Duponchel C., Carugati A., Choquet S., Agostoni A. (2000). Frequent *de novo* mutations and exon deletions in the C1 inhibitor gene of patients with angioedema. J Allergy Clin Immunol.

[28] Khan D.A. (2011). Hereditary angioedema: historical aspects, classification, pathophysiology, clinical presentation, and laboratory diagnosis. Allergy Asthma Proc.

[29] Zuraw B.L., Busse P.J., White M., Jacobs J., Lumry W., Baker J. (2010). Nanofiltered C1 inhibitor concentrate for treatment of hereditary angioedema. N Engl J Med.

[30] Craig T.J., Levy R.J., Wasserman R.L., Bewtra A.K., Hurewitz D., Obtulowicz K. (2009). Efficacy of human C1 esterase inhibitor concentrate compared with placebo in acute hereditary angioedema attacks. J Allergy Clin Immunol.

[31] Cicardi M., Bork K., Caballero T., Craig T., Li H.H., Longhurst H. (2012). Evidence-based recommendations for the therapeutic management of angioedema owing to hereditary C1 inhibitor deficiency: consensus report of an international working group. Allergy.

[32] Craig T., Pursun E.A., Bork K., Bowen T., Boysen H., Farkas H. (2012). WAO guideline for the management of hereditary angioedema. World Allergy Organ J.

