# Supplementary material for: Hereditary angioedema diagnosis evaluation score (HADES): A new clinical scoring system for predicting hereditary angioedema with C1 inhibitor deficiency
Source: J Allergy Clin Immunol Glob. 2025 Jan 17;4(2):100414. doi: 10.1016/j.jacig.2025.100414 (PMC11847109; doi:10.1016/j.jacig.2025.100414)
Supplement: Supplementary Figs and Tables [file mmc1.docx]

**Supplementary Figure 1.** Guidance questionnaire for suspected HAE cases (Spanish).

| 1- Edema recurrente |  | |  | |
| --- | --- | --- | --- | --- |
| Edema pruriginoso | Si | | No | |
| Edema en las extremidades | Si | | No | |
| Edema facial | Si | | No | |
| Persiste ≥24 horas | Si | | No | |
| Responde a antihistamínicos y/o corticoides | Si | | No | |
| 2- Ronchas con picazón | Si | | No | |
| 3- Dolor abdominal | Si | | No | |
| 4- Obstrucción intestinal que requirió cirugía | Si | | No | |
| 5- Vómitos | Si | | No | |
| 6- Edema de laríngeo | Si | | No | |
| 7- Eritema sin picazón | Si | | No | |
| 8- Síntomas causados por | Trauma | Menstruación | | Medicación |
|  | Infección | Estrés | | Sin causa |
| 9- Edad de inicio de síntomas (años) | 0 – 5 | 5 – 10 | | 10 – 20 |
|  | 20 – 30 | 30 – 40 | | 40 – 50 |
| 10- Ubicación de los síntomas |  | | | |
| 11- Historia familiar de angioedema | Si | | No | |
|  | Explicación: | | | |

Original questionnaire in Spanish.

**Supplementary Figure 2.** Stacked column chart showing the percentage of diagnosed and non-diagnosed suspected index cases by each clinical diagnostic score of HAE


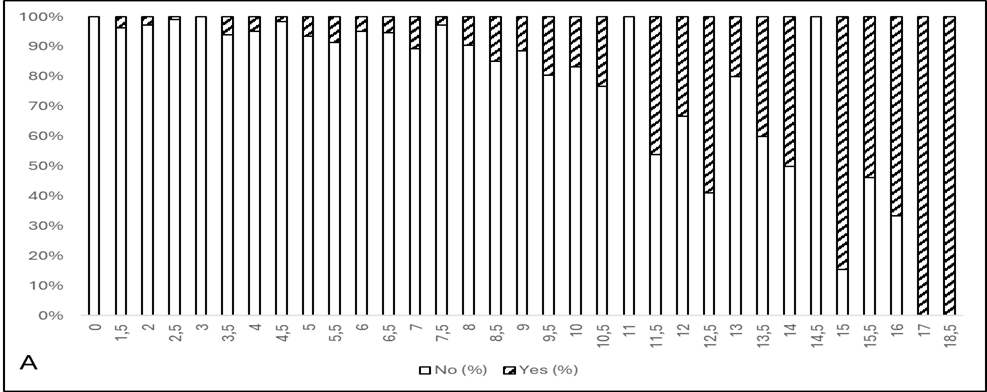


**Supplementary Figure 3.** Stacked column chart showing the percentage of diagnosed and non-diagnosed family cases by each clinical diagnostic score of HAE


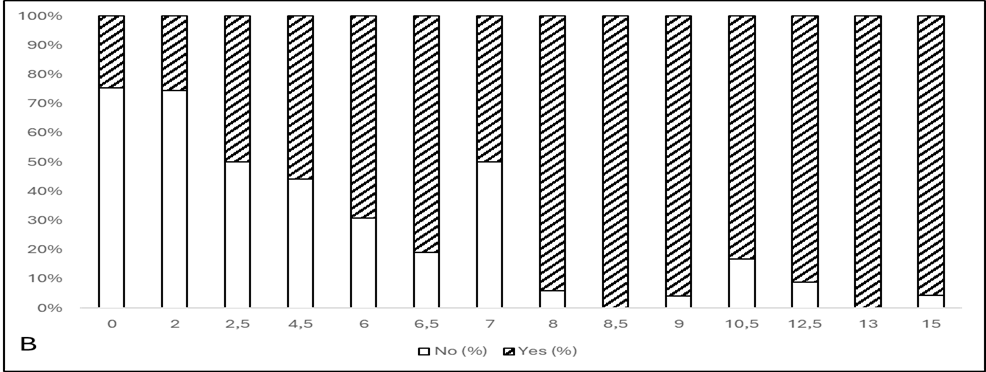


**Supplementary Table 1.** Internal validation in suspected index cases (bootstrapping).

|  | **ẞ** | **p value** | **95% CI of ẞ** | |
| --- | --- | --- | --- | --- |
|  |  |  | **Lower** | **Upper** |
| Age of onset ≤20 years | 0,537 | 0,013 | 0,124 | 0,958 |
| Recurrent limb edema | 0,894 | 0,001 | 0,458 | 1,380 |
| Abdominal pain | 0,635 | 0,002 | 0,211 | 1,069 |
| Family history of angioedema | 1,128 | 0,001 | 0,690 | 1,562 |
| Vomiting | 1,211 | 0,001 | 0,777 | 1,656 |
| Trauma as a trigger of symptoms | 1,262 | 0,001 | 0,736 | 1,765 |
| Absence of wheals | 0,927 | 0,001 | 0,553 | 1,364 |

**Supplementary Table 2.** Internal validation in family cases (bootstrapping).

|  | **ẞ** | **p value** | **95% CI of ẞ** | |
| --- | --- | --- | --- | --- |
|  |  |  | **Lower** | **Upper** |
| Recurrent limb edema | 1,506 | 0,001 | 1,017 | 2,060 |
| Recurrent edema ≥24 hours | 1,806 | 0,001 | 1,209 | 2,599 |
| Abdominal pain | 0,633 | 0,007 | 0,163 | 1,097 |
| Trauma as a trigger of symptoms | 0,921 | 0,017 | 0,198 | 1,842 |

**Supplementary Table 3.** Number of events per score in the suspected index cases.

|  | **Confirmed HAE diagnosis** | |  |
| --- | --- | --- | --- |
| **Score** | **No (n, %)** | **Yes (n, %)** | **Total** |
| 0 | 56 (100%) | 0 (0%) | 56 |
| 1.5 | 79 (96.3%) | 3 (3.7%) | 82 |
| 2 | 33 (97.1%) | 1 (2.9%) | 34 |
| 2.5 | 200 (99%) | 2 (1%) | 202 |
| 3 | 7 (100%) | 0 (0%) | 7 |
| 3.5 | 31 (93.9%) | 2 (6.1%) | 33 |
| 4 | 212 (95.1%) | 11 (4.9%) | 223 |
| 4.5 | 113 (98.3%) | 2 (1.7%) | 115 |
| 5 | 86 (93.5%) | 6 (6.5%) | 92 |
| 5.5 | 32 (91.4%) | 3 (8.6%) | 35 |
| 6 | 140 (95.2%) | 7 (4.8%) | 147 |
| 6.5 | 87 (94.6%) | 5 (5.4% | 92 |
| 7 | 83 (89.2%) | 10 (10.8%) | 93 |
| 7.5 | 36 (97.3%) | 1 (2.7%) | 37 |
| 8 | 47 (90.4%) | 5 (9.6%) | 52 |
| 8.5 | 57 (85.1%) | 10 (14.9%) | 67 |
| 9 | 23 (88.5%) | 3 (11.5%) | 26 |
| 9.5 | 62 (80.5%) | 15 (19.5%) | 77 |
| 10 | 15 (83.3%) | 3 (16.7%) | 18 |
| 10.5 | 23 (76.7%) | 7 (23.3%) | 30 |
| 11 | 1 (100%) | 0 (0%) | 1 |
| 11.5 | 7 (53.8%) | 6 (46.2%) | 13 |
| 12 | 22 (66.7%) | 11 (33.3%) | 33 |
| 12.5 | 7 (41.2%) | 10 (58.8%) | 17 |
| 13 | 8 (80%) | 2 (20%) | 10 |
| 13.5 | 3 (60%) | 2 (40%) | 5 |
| 14 | 4 (50%) | 4 (50%) | 8 |
| 14.5 | 1 (100%) | 0 (0%) | 1 |
| 15 | 2 (15.4%) | 11 (84.6%) | 13 |
| 15.5 | 6 (46.2%) | 7 (53.8%) | 13 |
| 16 | 1 (33.3%) | 2 (66.7%) | 3 |
| 17 | 0 (0%) | 1 (100%) | 1 |
| 18.5 | 0 (0%) | 6 (100%) | 6 |
| **Total** | **1484 (90.4%)** | **158 (9.6%)** | **1642** |

**Supplementary Table 4.** Number of events per score in the family cases.

| \|  \| \| --- \| | **Confirmed HAE diagnosis** | |  |
| --- | --- | --- | --- | --- |
| **Score** | **No (n, %)** | **Yes (n, %)** | **Total** |
| 0 | 356 (75.2%%) | 117 (24.7%) | 473 |
| 2 | 41 (74.5%) | 14 (25.5%) | 55 |
| 2.5 | 1 (50%) | 1 (50%) | 2 |
| 4.5 | 15 (44.1%) | 19 (55.9%) | 34 |
| 6 | 4 (30.8%) | 9 (69.2%) | 13 |
| 6.5 | 9 (19.1%) | 38 (80.9%) | 47 |
| 7 | 3 (50%) | 3 (50%) | 6 |
| 8 | 1 (5.9%) | 16 (94.1%) | 17 |
| 8.5 | 0 (0%) | 1 (100%) | 1 |
| 9 | 1 (4%) | 24 (96%) | 25 |
| 10.5 | 4 (16.7%) | 20 (83.3%) | 24 |
| 12.5 | 3 (8.8%) | 31 (91.2%) | 34 |
| 13 | 0 (0%) | 5 (100%) | 5 |
| 15 | 2 (4.4%) | 43 (95.6%) | 45 |
| **Total** | **440 (56.3%)** | **341 (43.7%)** | **781** |
